# Highly Pathogenic Avian Influenza Virus Infection of Mallards with Homo- and Heterosubtypic Immunity Induced by Low Pathogenic Avian Influenza Viruses

**DOI:** 10.1371/journal.pone.0006706

**Published:** 2009-08-20

**Authors:** Sasan R. Fereidouni, Elke Starick, Martin Beer, Hendrik Wilking, Donata Kalthoff, Christian Grund, Rafaela Häuslaigner, Angele Breithaupt, Elke Lange, Timm C. Harder

**Affiliations:** 1 Friedrich-Loeffler-Institut (FLI), Insel Riems, Germany; 2 Friedrich-Loeffler-Institut (FLI), Wusterhausen, Germany; London School of Hygiene & Tropical Medicine, United Kingdom

## Abstract

The potential role of wild birds as carriers of highly pathogenic avian influenza virus (HPAIV) subtype H5N1 is still a matter of debate. Consecutive or simultaneous infections with different subtypes of influenza viruses of low pathogenicity (LPAIV) are very common in wild duck populations. To better understand the epidemiology and pathogenesis of HPAIV H5N1 infections in natural ecosystems, we investigated the influence of prior infection of mallards with homo- (H5N2) and heterosubtypic (H4N6) LPAIV on exposure to HPAIV H5N1. In mallards with homosubtypic immunity induced by LPAIV infection, clinical disease was absent and shedding of HPAIV from respiratory and intestinal tracts was grossly reduced compared to the heterosubtypic and control groups (mean GEC/100 µl at 3 dpi: 3.0×10^2^ vs. 2.3×10^4^ vs. 8.7×10^4^; p<0.05). Heterosubtypic immunity induced by an H4N6 infection mediated a similar but less pronounced effect. We conclude that the epidemiology of HPAIV H5N1 in mallards and probably other aquatic wild bird species is massively influenced by interfering immunity induced by prior homo- and heterosubtypic LPAIV infections.

## Introduction

Migratory birds and members of the *Anseriformes* order in particular, have been suspected as carriers of highly pathogenic avian influenza virus (HPAIV) subtype H5N1 from Southeast Asia into central Asia, Europe and Africa. The primary occurrence of the infection in wild birds in several countries and rapid westward spread of HPAIV H5N1 in 2005 and 2006 have sparked such assumptions [1]. However, the role of wild birds as culprits of H5N1 spread has been heavily debated. Instead, legal and illegal trading practices of poultry, poultry products and captive wild birds were put into focus [2], [3], [4].

Previous experimental studies with HPAIV H5N1 strains of different origins in various species of water birds including swans and geese [5], [6], [7], gulls [8], [9], [10] and ducks [9], [11], [12] showed that AIV seronegative swans, especially black swans (*Cygnus atratus*), Canada geese (*Branta canadensis*) and laughing gulls (*Larus atriculla*) are highly vulnerable to H5N1 infection. Diving ducks including wood ducks (*Aix sponsa*) and pochards (*Aythya ferina*) were also found susceptible, while dabbling ducks including northern pintails (*Anas acuta*), blue-wing teals (*Anas crecca*), redheads (*Aythya americana*) and mallards (*Anas platyrhynchos*) were less susceptible or tolerant [9]. In these studies the potential role of the latter species which include the most prevalent Eurasian wild duck species, the mallard, for long-distance spread of H5N1 virus was stressed [11], [12]. However, there is only a single report of healthy wild duck (common pochard in Switzerland) found in Europe to be naturally infected by HPAIV H5N1 although several clustered outbreaks of symptomatic influenza among wild birds in Europe, some involving mallards, have occurred [13], [14]. Sample sizes in cross-sectional surveys of wild birds may not have been large enough to exclude a prevalence of approximately less than 1% of HPAIV H5N1. Outbreaks among wild birds, nevertheless, proved to be limited in temporal and geographical extension as well as in numbers of individual birds infected. In Germany, in 2006 only 344 wild birds, mainly swans and geese, were found dying of an HPAIV H5N1 infection despite presence of several hundred thousand individuals of these species in the same area [13]. The reasons for this observation are still not clear, but it is likely that not all infected individual birds develop symptomatic influenza.

It has been hypothesized that a considerable number of these birds may have at least partially been protected by immunity induced by naturally occurring homosubtypic (HA homologous) infection with avian influenza viruses of low pathogenicity (LPAIV), and that cross reactive interference of even heterosubtypic (HA heterologous) LPAIV-induced immunity might have played a silencing role. LPAIV H5 strains are being continuously isolated from *Anatidae* species including mallards. AIV prevalence in wild ducks along the southern coasts of the North and the Baltic Seas can reach 14% during autumn migration [15]. However, no reliable seroprevalence data from wild *Anatidae* exist to support this assumption.

We tested the effect of LPAIV-induced immunity by experimental inoculation of seronegative (for whole period before inoculation) captive mallards, with two different LPAIV subtypes, H5N2 and H4N6, and subsequent challenge infection with HPAIV H5N1. An H4 subtype virus was chosen because (i) the HA of this subtype is distantly related, by genetic and antigenic means, to that of the H5 subtype and (ii) subtype H4 viruses show a high prevalence in wild duck populations. Mallards represent the most abundant duck species in Eurasia and migrate over long distances, e.g., along the East-Atlantic flyway [16]. In our study, we provide evidence that both LPAIV-induced homo-subtypic and heterosubtypic (H4) immunity modulate, to a different extent, H5N1 excretion in mallards.

## Materials and Methods

### Viruses

The three AIV strains used in this study are maintained in the virus repository of the OIE and National Reference Laboratory for Avian Influenza (NRL AI) at the Friedrich-Loeffler-Institut (FLI). The LPAIV strains A/mallard/Föhr (Germany)/Wv1806-09K/03(H4N6) and A/duck/Potsdam/1402/86(H5N2) were used for pre-exposure inoculation of ducks. The HPAIV strain A/duck/Vietnam/TG24-01/05(H5N1) was used for challenge infection. This clade 1 isolate bears a PQRERRKKR/GLF motif at the HA_0_ cleavage site, and has an intravenous pathogenicity index (IVPI) of 2.9 in specific pathogen free (SPF) chickens; in addition, it has been found to induce clinically overt and lethal neurological disease in adult Pekin ducks (Harder et al., unpublished).

### Experimental design

Thirty–two mallards (*Anas platyrhynchos*) were captive-bred and housed indoors in the quarantine building of the FLI. The birds were handled and cared for in accordance with the Animal Protection guidelines and legal approval (trial approval LVL M-V/TSD/7221.3-1.1-003/07). All experiments with HPAIV were conducted under Biosafety Level 3–agriculture (BSL-3-Ag) conditions. At 12 weeks of age, 24 ducks were transported to Biosafety Level 3 (BSL-3) facilities at the FLI. The ducks were inoculated with LPAI viruses, after one week of acclimatization, when they were 13 weeks of age. At this age juvenile free-ranging mallards reveal highest prevalence of LPAIV infections, which is consistent with pre-migration staging in the late summer or early fall [9], [17]. Prior to inoculation, oropharyngeal and cloacal swabs were collected from each bird to ensure they were not infected with any subtype of avian influenza virus at the start of the study. In addition, serum samples had been collected regularly since week 4 of age to confirm they were continuously AIV-negative by NP-specific antibody testing with competitive enzyme linked immuno-sorbent assay (ELISA) and haemagglutinin inhibition (HI) test using H4 and H5 subtype antigens. The ducks were randomly assembled into two experimental groups (male and female ducks were included in each group in approximately equal numbers) and each group was housed separately in self-contained isolation units, including: *H4 group:* twelve ducks which were inoculated via ocular, nasal and oropharyngeal routes with one millilitre (10^6^ EID_50_) of the H4N6 strain; *H5 group:* twelve ducks inoculated in the same way and the same doses with the H5N2 strain; *Control group:* eight ducks stayed in quarantine until challenge. All birds were continuously monitored for clinical symptoms and blood samples were collected at 1, 2, 4 and 7 weeks after LPAIV inoculation. Serum samples were tested by ELISA and HI tests.

Seven weeks after LPAIV inoculation, all 32 birds (including controls) were housed together in a BSL-3-Ag facility at the FLI. Oropharyngeal and cloacal swabs were collected from each bird to exclude active infection with and shedding of H4 or H5 LPAIV. Subsequently, all birds were challenged with 10^5^ TCID_50_ of HPAIV H5N1 strain via ocular, nasal and oropharyngeal routes. The birds were then monitored daily for clinical signs of disease. Oropharyngeal and cloacal swabs were collected from all birds at 1, 2, 3, 4, 7, 10, 14 and 21 days post challenge (dpc) and tested by real-time RT-PCR. The experiment was terminated at 24 dpc when serum samples were collected for serological testing and tissue samples including brain, lungs, liver and pancreas were obtained for virological evaluation.

Hypothesis test of differences between groups are carried out by a Mann-Whitney-U-Test in R.

### Real-time reverse transcription-PCR (RT-PCR)

**Figure 1:**
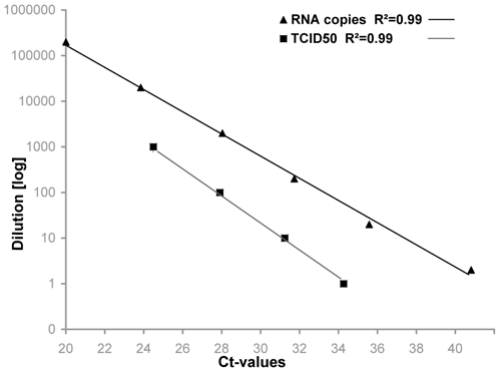
Regression analysis for the calibration of the genome equivalent RNA copy numbers (triangles) and the tissue culture infectious doses 50% endpoints (squares). Log_10_ dilution series of quantified RNA run-off transcripts or replication-competent H5N1 virus (infectivity titrated on MDCK cell culture) were used.

Swab and tissue samples were tested with TaqMan one-step real-time RT-PCR assays targeting the influenza A virus M gene [18] and an H5 subtype gene fragment [19] using the SuperScript III One-step RT-PCR kit with Platinum Taq DNA polymerase (Invitrogen) on a MX3000P Real-Time PCR System (Stratagene). In all tests, negative RNA preparation controls, and negative and positive RT-PCR controls as well as an internal transcription and amplification control (IC-2) were included [20]. The number of viral M gene copies or genome equivalent copy numbers (GEC) in 100 µl of the swab samples fluid was determined on basis of calibration experiments using RNA run-off transcripts of a plasmid carrying the M gene fragment under control of a T7 promoter (Figure 1).

### Hemagglutination inhibition (HI) assay

The hemagglutination inhibition (HI) assay was performed as previously described [19] using four hemagglutination units of inactivated antigen prepared from AIV strains A/mallard/Föhr (Germany)/Wv1806-09K/03 (H4N6), A/duck/Potsdam/1402/86 (H5N2) and A/duck/Vietnam/TG24-01/05 (H5N1).

### Competitive ELISA

The serum samples were tested with a competitive ELISA targeting influenza A nucleoprotein antibodies following the manufacturer's instructions (ID Screen, Influenza A NP Antibody Competition, ID.VET).

### Serum neutralisation test

The serum samples of all ducks have been tested by serum neutralization test (SNT), to quantify the serologic response, based on a previously described procedure [21].

### Virus titration

The titre of HPAIV in the swab samples was extrapolated from Ct-values on basis of calibration experiments using different log_10_ dilution series of A/duck/Vietnam/TG24-01/05 (H5N1) virus. Infectivity is expressed as TCID_50_ per 100 µl of the swab sample fluids (Figure 1).

### Immunohistochemistry and pathology

Tissues samples including trachea, lungs, heart, cerebrum, cerebellum, spinal cord, proventriculus, gizzard, small and large intestine, liver, pancreas and kidney of two birds from the control group, which died at 5 and 6 dpc, were collected, formalin fixed and processed for paraffin embedding according to standard procedures, and immunohistochemistry for influenza virus A nucleoprotein (NP) was performed. Briefly, after dewaxing sections were microwave irradiated for antigen retrieval (2×5 min, 600 W, 10 mM citrate buffer pH 6.0) and were incubated with a rabbit anti-NP serum (1∶750). A biotinylated goat anti-rabbit IgG1 (Vector, Burlingame, CA, USA) was applied (1∶200) as secondary antibody. By means of the avidin-biotin-peroxidase complex method, a bright red intracytoplasmatic and nuclear signal was observed. Positive control tissues of chickens experimentally infected with HPAI virus (H5N1) and additionally, a control primary rabbit serum against bovine papillomavirus (BPV 1∶2000) were included.

## Results

### Status before LPAIV exposure

The cloacal and oropharyngeal swab samples collected from 32 ducks during 8 weeks prior to LPAIV inoculation revealed negative results by real-time RT-PCR, indicating that the ducks were not shedding AIV before experimental infection. In addition, ducks were serologically negative to influenza A antigens tested by ELISA and HI tests (with using H4N6, H5N2 and H5N1 antigens), indicating that the birds were not exposed to AIV before inoculation.

### LPAIV infection and status before HPAIV challenge

All birds remained clinically healthy during seven weeks after inoculation of H4 and H5 LPAIV. The results of serological evaluation of ducks by ELISA and HI tests using the homologous antigens at 1, 2, 4 and 7 weeks after inoculation are summarized in Table 1. Serum samples from the control group were serologically negative when tested by ELISA, HI and serum neutralization tests. The cloacal and oropharyngeal swab samples collected from all ducks before challenge, were negative in real-time RT-PCR indicating no virus shedding before HPAIV inoculation.

**Table 1:** The serological status of ducks inoculated by LPAI H4 and H5 viruses and challenged by HPAIV H5N1 as evaluated by competitive ELISA, HI and serum neutralisation tests.

Group	ELISA 1						H4N6 HI 2					H5N2-HI					H5N1-HI	SN-test 3	
	B.I. 4	1*	2	4	7	Post C.	B.I.	1	2	4	7	B.I.	1	2	4	7	Post C.^5^	Before C.	Post C.
H5	0/12	12/12	12/12	7/12	6/12	12/12	<2	<2 6	<2	<2	<2	<2	5.1±0.8	5.8±0.8	5.7±0.5	4±0.4	6.7±1.1	5.4	9.5
H4	0/12	12/12	12/12	9/12	3/12	12/12	<2	2.6±2.2	4.3±1.4	4±0.9	2.3±1	<2	<2	<2	<2	<2	7.2±0.9	2	8.4
Control	0/8	0/8	0/8	0/8	0/8	7/7	<2	<2	<2	<2	<2	<2	<2	<2	<2	<2	8.4±0.9	<2	8.7

* The numbers indicate weeks after LPAIV inoculation.

1 The ELISA results indicated as the number of positives out of the number of tested birds.

2 Hemagglutination inhibition results indicate the geometric mean titre of (log_2_) serum samples and mean log_2_±standard deviation.

3 Carried out against AI virus A/cygnus cygnus/Germany/R65/06 (H5N1) and expressed as geometric mean titre of (log_2_) serum samples.

4 Before LPAIV H4N6 or H5N2 inoculation.

5 Post C. = 24 days post challenge. Before C. = the day of challenge, before inoculation of HPAIV.

6 None of the ducks with mean titre of <2, showed reactivity higher than 1 log_2._

### HPAIV challenge infection

#### Clinical symptoms

Clinical symptoms varied significantly among members of the three groups. From day two after inoculation onwards, up to seven ducks in the control group became severely sick, but only one of the control birds died (6 dpc) while others recovered slowly. One more duck died at 5 dpc. Unfortunately due to loss of the wing tag of this bird and also another bird from the H4 group at the same day, it could not be unambiguously assigned to either H4 or control groups (see also footnote 6 in Table 2). Clinical signs included severe weakness, loss of appetite, mild diarrhea and listlessness. Neurological signs mainly consisting of neck tremor were evident in one of the control ducks. Three out of 12 ducks from the H4 group transiently showed mild clinical symptoms consisting of listlessness and loss of appetite. In one duck of this group unilaterally a cloudy eye was evident. No clinical signs were observed in the H5 group.

**Table 2:** Real-time RT-PCR results (based on 15 µl swab fluids), GEC values and TCID_50_ (both based on 100 µl swab fluids) of tracheal and cloacal samples obtained from ducks after challenge with HPAIV H5N1 strain.

Group		Cloacal swabs (dpc)								Tracheal swabs (dpc)							
		1*	2	3	4	7	10	14	21	1	2	3	4	7	10	14	21
H5	Pos. No. 1	0/12	0/12	0/12	0/12	0/12	0/12	0/12	0/12	3/12	3/12	2/12	1/12	0/12	0/12	0/12	0/12
	Average Ct 2	neg	neg	neg	neg	neg	neg	neg	Neg	34.8	34.07	34.25	34.24	neg	neg	neg	neg
	Ct Range^3^	-	-	-	-	-	-	-	-	34.2–35.1	33.5–34.8	33.3–35.3	-	-	-	-	-
	Average GEC 4	<1	<1	<1	<1	<1	<1	<1	<1	1.1×10^3^	1.7×10^3^	1.8×10^3^	1.5×10^3^	<1	<1	<1	<1
	GEC range 5	-	-	-	-	-	-	-	-	9.0×10^2^–1.6×10^3^	1.1×10^3^–2.4×10^3^	8.0×10^2^–1.8×10^3^	-	-	-	-	-
	Average TCID_50_	<1	<1	<1	<1	<1	<1	<1	<1	1.0×10^1^	1.3×10^1^	1.7×10^1^	1.5×10^3^	<1	<1	<1	<1
	TCID_50_ Range	-	-	-	-	-	-	-	-	7.0–1.3×10^1^	7.0–2.0×10^1^	7.0–2.6×10^1^	-	-	-	-	-
H4	Pos. No.	0/12	0/12	0/12	0/12	0/11 7	0/11	0/11	0/11	12/12	12/12	12/12	10/12	4/11	1/11	0/11	0/11
	Average Ct	neg	neg	neg	neg	Neg	neg	neg	Neg	29.84	29.8	30.67	32.39	33.99	34.16	neg	neg
	Ct Range	-	-	-	-	-	-	-	-	24.2–34.5	24.1–33	27–33.2	29.8–35.1	33.2–35.1	-	-	-
	Average GEC	<1	<1	<1	<1	<1	<1	<1	<1	7.5×10^4^	7.1×10^4^	2.3×10^4^	8.6×10^3^	1.9×10^3^	1.6×10^3^	<1	<1
	GEC Range	-	-	-	-	-	-	-	-	1.3×10^3^–5.8×10^5^	3.1×10^3^–6.1×10^5^	2.9×10^3^–1.1×10^5^	8.9×10^2^–2.1×10^4^	8.9×10^2^–2.8×10^3^	-	-	-
	Average TCID_50_	<1	<1	<1	<1	<1	<1	<1	<1	1.4×10^3^	1.3×10^3^	3.1×10^2^	7.5×10^1^	1.7×10^1^	1.3×10^1^	<1	<1
	TCID_50_ Range	-	-	-	-	-	-	-	-	1.3×10^1^–1.2×10^4^	2.6×10^1^–1.3×10^4^	2.6×10^1^–1.8×10^3^	7.0×10^0^–2.4×10^2^	7.0×10^0^–2.6×10^1^	-	-	-
Control	Pos. No.	4/8	4/8	6/8	4/8	1/6 6	0/6	0/6	0/6	8/8	8/8	8/8	8/8	6/6	4/6	2/6	1/6
	Average Ct	33.91	32.71	32.15	32.17	35.2	neg	neg	Neg	28.12	27.54	27.83	29.44	33.26	33.9	34.45	34.3
	Ct Range	32.7–34.8	30.33–34.37	26.9–34.6	28.4–35.2	-	-	-	-	22.7–33.6	24.6–31.6	26.3–30.1	27.0–31.4	29.3–35.6	31.8–34.4	34.0–34.9	-
	Average GEC	2.1×10^3^	5.9×10^3^	2.2×10^4^	1.6×10^4^	8.6×10^2^	<1	<1	<1	2.5×10^5^	1.4×10^5^	8.7×10^4^	3.6×10^4^	5.8×10^3^	2.9×10^3^	1.4×10^3^	1.5×10^3^
	GEC range	1.1×10^3^–3.9×10^3^	1.8×10^3^–1.5×10^4^	1.2×10^3^–1.1×10^5^	8.2×10^2^–4.9×10^4^	-	-	-	-	2.1×10^3^–1.4×10^6^	7.3×10^3^–4.6×10^5^	1.8×10^4^–1.7×10^5^	8.2×10^3^–1.1×10^5^	6.6×10^2^–2.8×10^4^	7.3×10^2^–6.6×10^3^	1.0×10^3^–1.7×10^3^	-
	Average TCID_50_	1.8×10^1^	6.1×10^1^	3.3×10^2^	2.1×10^2^	7.0×10^0^	<1	<1	<1	5.7×10^3^	2.5×10^3^	1.4×10^3^	5.0×10^2^	6.8×10^1^	2.9×10^1^	1.0×10^1^	1.1×10^1^
	TCID_50_ range	7.0×10^0^–3.3×10^1^	1.3×10^1^–1.7×10^2^	2.0×10^1^–1.8×10^3^	1.0×10^1^–6.7×10^2^	-	-	-	-	2.1×10^1^–3.4×10^4^	7.3×10^1^–9.4×10^3^	2.1×10^2^–2.9×10^3^	7.9×10^1^–1.7×10^3^	1.3×10^1^–3.5×10^2^	1.3×10^1^–6.6×10^1^	7.0×10^0^–1.3×10^1^	-

* The numbers indicate days post-challenge (dpc).

1 Number of positive birds as indicated by real-time RT-PCT. Ct-values<36 considered as positive.

2 Average ct-value of positive ducks per 15 µl of swab fluid.

3 The max and min range of positive ct-values.

4 Average genome equivalent copy (GEC) number of positive samples/100 µl of swab fluid.

5 Average tissue culture infectious dose (TCID_50_) titre/100 µl of swab fluid.

6 At this stage one of the control birds died.

7 Two ducks lost the wing tags at 5 dpc, and one of them died at 5 dpc, since their assignment to either H4 or control groups was not possible, both were excluded from calculations after day 5.

#### Respiratory & intestinal viral shedding

**Figure 2:**
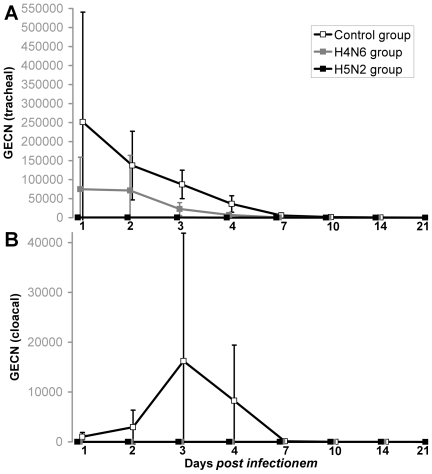
Excretion of HPAIV H5N1 viral RNA via the respiratory (A) or intestinal (B) tracts as measured by rRT PCR targeting a M gene fragment in three groups of experimentally challenged mallards. Average of virus excretion and 95% confidence intervals are depicted.

The results of the real-time RT-PCR testing of cloacal and oropharyngeal swab samples taken on days 1, 2, 3, 4, 7, 10, 14, 21 after challenge are summarized in Figure 2 and Table 2. Cloacal and oropharyngeal excretion of HPAIV (H5N1) varied significantly among the three groups. In general, oropharyngeal excretion was much more pronounced. In the control group, viral shedding from the respiratory tract started at 1 dpc and continued with high viral genome loads for four days (on average 2.5×10^5^, 1.4×10^5^, 8.7×10^4^ and 3.6×10^4^ GEC per 100 µl of swab fluid for the first four days which relates to infectivity titres of 5.6×10^3^, 2.5×10^3^, 1.4×10^3^ and 5.1×10^2^ TCID_50_ in 100 µl of swab fluid, respectively; Table 2). During these days also peaks of clinical signs were observed. Shedding continued in seven and four control ducks, respectively, until 7 and 10 dpc (Table 2). Two ducks from the control group continued respiratory viral shedding at low virus genome loads for two weeks and one duck continued for three weeks post-challenge, even after recovering from clinical disease (Table 2). The ducks of the control group also excreted virus from the intestinal tract during the first week after infection, but at lower average genome loads (mean GEC/100 µl at 3 dpi: 8.7×10^4^ vs. 2.2×10^4^; p<0.05) and also for a shorter time compared to oropharyngeal swabs (Figure 2, Table 2). All cloacal samples were negative after one week post challenge infection.

Cloacal shedding was not observed in ducks from groups with previous LPAIV infection. Clear differences were also seen regarding the oropharyngeal shedding of the H4 and H5 groups, especially on day 3 and 4 significant differences in tracheal shedding is observed between all three groups (Figure 2). Whereas in the H4 group viral genome loads of 7.5×10^4^, 7.1×10^4^ and 2.3×10^4^ GEC/100 µl (equal to 1.4×10^3^, 1.3×10^3^ and 3.1×10^2^ TCID_50_) were observed during the first three dpc, samples from only three ducks of the H5 group with loads less than 1.8×10^3^ GEC/100 µl were found positive (Table 2). The viral genome loads were higher and lasted for longer periods in oropharyngeal swabs of the control group than in samples of groups immunized with heterologous (H4) or homologous (H5) LPAIV respectively (mean GEC/100 µl 3 at dpi: 8.7×10^4^ vs. 2.3×10^4^ vs. 3.0×10^2^; p<0.05).

Tissue samples comprising brain, lung, liver and pancreas from the one control duck which died at 6 dpc were highly positive in real-time RT-PCR (2.0×10^7^, 5.1×10^4^, 4.4×10^4^ and 1.1×10^6^ GEC/100 µg, respectively). No viral RNA/infectivity was detected in the same tissues from any of the ducks surviving until 24 dpc.

#### Serological findings

Surviving ducks in all three groups developed high levels of HPAIV H5-specific antibodies post-challenge according to NP-ELISA, H5-specific HI and serum neutralization tests (Table 1). Serum neutralization titres against the challenge virus at 24 dpc ranged around 9 log_2_ (1∶512) in all three groups with no significant differences among them.

#### Pathological findings

**Figure 3:**
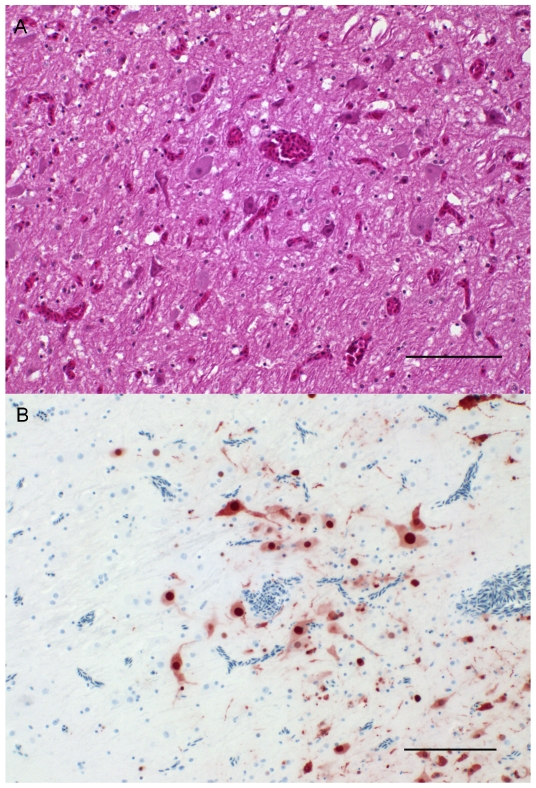
Histopathology and immunohistochemistry of brain from infected ducks. (A) Brain, Cerebrum; Duck at 5 dpc. Congestion shown by hematoxylin-eosin staining. Bar 100 µm. (B) Brain, Cerebrum; Duck at 6 dpc. Intense intranuclear and intracytoplasmic AIV antigen staining within neurons and neuroglia. Immunohistochemistry. ABC method using anti-NP monoclonal antibody HB65, hematoxylin counterstain. Bar 100 µm.

The control duck, which died at 6 dpc showed moderate congestion of the liver and edema of the brain. In histopathology, the cerebrum was severely congested, multifocally there was neuropil degeneration with mild vacuolation (Figure 3A) hemorrhage and glial nodules. Ventricles of the cerebrum were filled with blood, and a mild lymphoplasmacellular meningoencephalitis with few macrophages was present. Within the lungs there was a moderate congestion and edema. Besides this, severe heterophilic infiltrates, predominantly adjacent to parabronchi were observed. The heart and the liver showed mild multifocal parenchymal degeneration accompanied by lymphoplasma-histiocytic infiltrates. Influenza virus nucleoprotein was detected by immunohistochemistry within the brain (neurons and glial cells, Figure 3B), the liver (hepatocytes), the lung (bronchiolar epithelium and alveolar macrophages) and the heart (myocardiocytes). One more duck died at 5 dpc with abovementioned clinical signs, but due to loss of its wing tag, it could not be unambiguously assigned to either H4 or control groups (see also footnote 6 in Table 2). Ducks surviving until 24 dpc did not reveal any gross lesions.

## Discussion

Here we show that pre-existing immunity induced by infection with homo- or heterosubtypic LPAIV modifies the course of an experimental challenge infection with HPAIV H5N1 in mallards. Clinical signs as well as amplitude and tissue tropism of virus shedding was affected.

Seven (out of eight) control ducks became severely sick. In contrast, only three ducks (out of 12) with previous H4N6 infection showed mild clinical symptoms but recovered fast, and no clinical symptoms were obvious in ducks with previous H5N2 LPAIV infection. Viral shedding from the respiratory tract was most pronounced in control ducks. Preferential viral shedding via the oropharynx has been consistently demonstrated with HPAIV H5N1 viruses [9], [12], [22].Two controls ducks even continued viral shedding at low titres for two more weeks after resolution of clinical symptoms. Viral shedding in the H4 group was markedly shortened and at lower titres (3 and 4 dpc). Just a few ducks from the H5 group were shedding the virus at very low titres compared to the control group. Cloacal viral shedding was evident only in ducks of the control group.

Clinical symptoms in ducks of the control group seemed to be more severe that has previously been reported for experimental inoculation of naive mallards with HPAIV H5N1 [9], [23]. The observed variability in clinical symptoms and modes of oropharyngeal viral shedding among different studies could be due to virus strain-specific characteristics [22], [24], [25]. However, low level pre-existing AIV-specific immunity could explain the attenuation effect seen in some birds tested in previous studies.

Long-distance migration is one of the most demanding physiologic activities in the animal world [26] and although no overt clinical symptoms have been observed during previous experimental HPAIV infections of mallards, these birds may not have been able to engage on long distance migration flights at the height of viral infection. Previous experimental studies demonstrated that oropharyngeally excreted HPAIV originated from lung and air sacs [12], implicating a high replication rate of the virus and thus a possible functional impairment in organs important for long-distance flight and migration. From this point of view it seems more likely to assume that long distance transposition of HPAIV by migrating *Anatidae* might rather occur during the incubation period. This period may last only a few days. Nevertheless, in this study two control ducks shed virus for more than seven days after resolving of clinical signs of infection albeit at lower titers. Therefore, these birds may contribute to local transmission of the virus. Also, many ducks of the H4 group were shedding the virus, again at lower titers, in absence of clinical disease. Spread of virus by such individuals, at least over shorter to medium distances, can likewise not by excluded. Also, the high intra-species variability in susceptibility to HPAI (H5N1) viruses observed in many wild bird species during regional outbreaks in Europe in 2006 and 2007 may in fact be also explained by different levels of AIV-specific immunity primed by previous LPAIV infections.

In summary, the results of our study show that, in captive mallards, heterosubtypic cross reactive immunity can derogate clinical symptoms of an HPAIV H5N1 infection, reduce the amount and duration of viral shedding from the respiratory tract and prevent viral shedding from the intestinal tract. Homosubtypic immunity may fully abrogate clinical symptoms and viral shedding from the intestinal tract, and drastically reduce viral shedding from the respiratory tract. Therefore, mallards with prior exposure to homologous LPAI viruses may remain healthy and might be suitable for long-distance transposition of HPAIV, but probably only shed very low titers of virus. Mallards with prior exposure to heterosubtypic LPAI viruses might pose a greater risk for transmission and spread of HPAIV, because they can shed higher amounts of virus (but only via the respiratory route) without developing severe clinical disease. Still, the potential role of respiratory shedding compared to intestinal shedding in the efficacy of bird-to-bird transmission of HPAIV in the nature needs to be clarified.
